# Ischemic postconditioning diminishes intramyocardial hemorrhage in acute reperfused myocardial infarction in rats, evaluated by CMR at 7T

**DOI:** 10.1186/1532-429X-17-S1-M5

**Published:** 2015-02-03

**Authors:** Bing Zhang, Wei Chen, Yushu Chen, Fabao Gao

**Affiliations:** 1 West China Hospital, Sichuan University, Chengdu, China

## Background

Ischemic postconditioning (IPOC) is considered to be a beneficial tool which can reduce reperfusion injury in acute myocardial infarction (AMI). We aim to assess the effect of IPOC on intramyocardial hemorrhage in rats with AMI.

## Methods

Twenty Sprague-Dawley rats (female, 250-300g) were divided into two groups: control group and IPOC group. The AMI model was built by ligating the left anterior descending coronary artery. All rats were subjected to 60 min ischemia, after that control group received direct reperfusion and IPOC group received 1 min of postconditioning (3 cycles of 10s reperfusion and 10s reocclusion) before permanent reperfusion. 48 hours later, all the rats were underwent cardiac magnetic resonance imaging (CMR) at 7.0T (Bruker BioSpect70/30, Germany). Cine (TR/TE=5.2ms/2.0ms, FA=10°, MTX=192×192, FOV=50×50mm, slice thickness=1.5mm), Late gadolinium enhancement (TR/TE=5.2ms/1.8ms, FA=25°, MTX=256×256, FOV=50×50mm, slice thickness=1.5mm) and simplified T2-mapping (TR/TE=1500ms/10,20,30ms, MTX=192×192, FOV=50×50mm, slice thickness=1.5mm) were performed to acquire cardiac function indexes(EDV, ESV and EF), infarct size (LV%) and the area at risk (edematous area, LV%) respectively. All rats were sacrificed after CMR and the hearts were excised for hematoxylin-eosin staining. The results were expressed as mean ± SD and independent sample T-test was performed to determine difference between groups.

## Results

**Figure 1:**
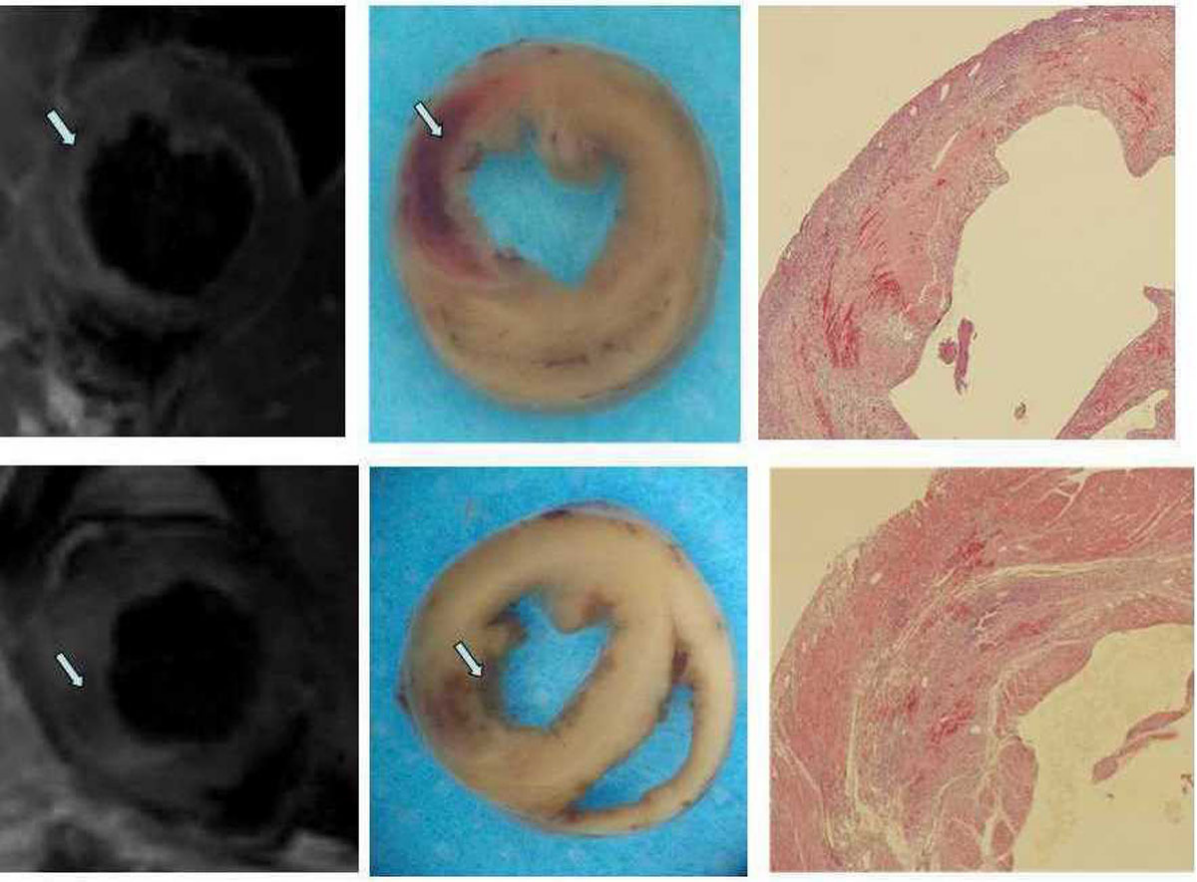
Intromyocardial hemorrhage in control group and IPOC group.

Sixteen rats left (Control=9; IPOC=7) for three rats were died during operation and one was excluded for image artifacts. Intramyocardial hemorrhage size was significantly larger in control group compared with IPOC group(9.3±2.6% vs. 2.4±0.3%, P = 0.00).There was no significant difference was observed in infarct size (46.7±13.1% vs. 37.7±12.1%, P =NS), area at risk (65±11.8% vs. 51.4±13.2%, P = NS) and EF(57.7±12.4% vs. 57.6±13.5%, P = NS). Figure 1

## Conclusions

IPOC may significantly reduce intramyocardial hemorrhage but cannot decrease myocardial edema and myocardial infarct size and it all failed to improve EF. It is known that intramyocardial hemorrhage is included as an aspect of reperfusion injury, so we can conclude that IPOC has a positive effect to dimimish reperfusion injury.

## Funding

This study was supported by The National Natural Science Foundation of China (81130027).

